# Analysis of the coal seam spalling–failure mechanism based on the seepage instability theory

**DOI:** 10.1371/journal.pone.0219735

**Published:** 2019-07-18

**Authors:** Hengjie Qin, Jianping Wei, Sen Li

**Affiliations:** 1 School of Architectural and Environmental Engineering, Zhengzhou University of Light Industry, Zhengzhou, Henan, China; 2 School of Safety Science and Engineering, Henan Polytechnic University, Jiaozuo, Henan, China; China University of Mining and Technology, CHINA

## Abstract

Coal and gas outburst is a common coal-rock dynamic disaster. Such accidents frequently occur, and the mechanism underlying the occurrence of these outbursts is complex. As a typical failure mode of a gas-filled and pressure-relieved coal body, the spallation mechanism should be investigated to reveal the mechanism of coal and gas outburst and guide outburst-prevention strategies. In this paper, a fluid-solid coupling model for coal seam gas flow is established. This model considers the adsorption characteristics of coal. Numerical calculations are used to simulate the stress field distribution and evolution of gas-filled coal bodies under different boundary conditions. The mechanical mechanism of the spallation occurrence after the pressure relief of coal is explained from the perspective of seepage breaking coal. The control of the flow and stress state of the gas to the spallation failure is analyzed. The mechanical-quantitative conditions for the initial failure of the coal body under seepage and the mechanical-qualitative conditions for the continuous advancement and termination of spallation are studied based on numerical solution results. The numerical calculation results show that the formation of a flow field after pressure relief will apply a drag force (tensile stress) on the porous media of coal. The presence of this force plays a crucial role in promoting the spallation and cracking of coal and, thus, the promotion of spallation. The tensile strength, initial adsorption pressure, and pressure relief rate of the coal body jointly control whether the initial failure can occur and the thickness of the fracture layer cracks. Spallation propulsion is mainly determined by the pressure relief conditions of the undestroyed coal body and pressure changes in the spallation space; the former can be quantitatively obtained by numerical calculations, whereas the latter is related to the thickness of the spalled layer and the degree of the layer-crack structure.

## Introduction

**Fig 1:**
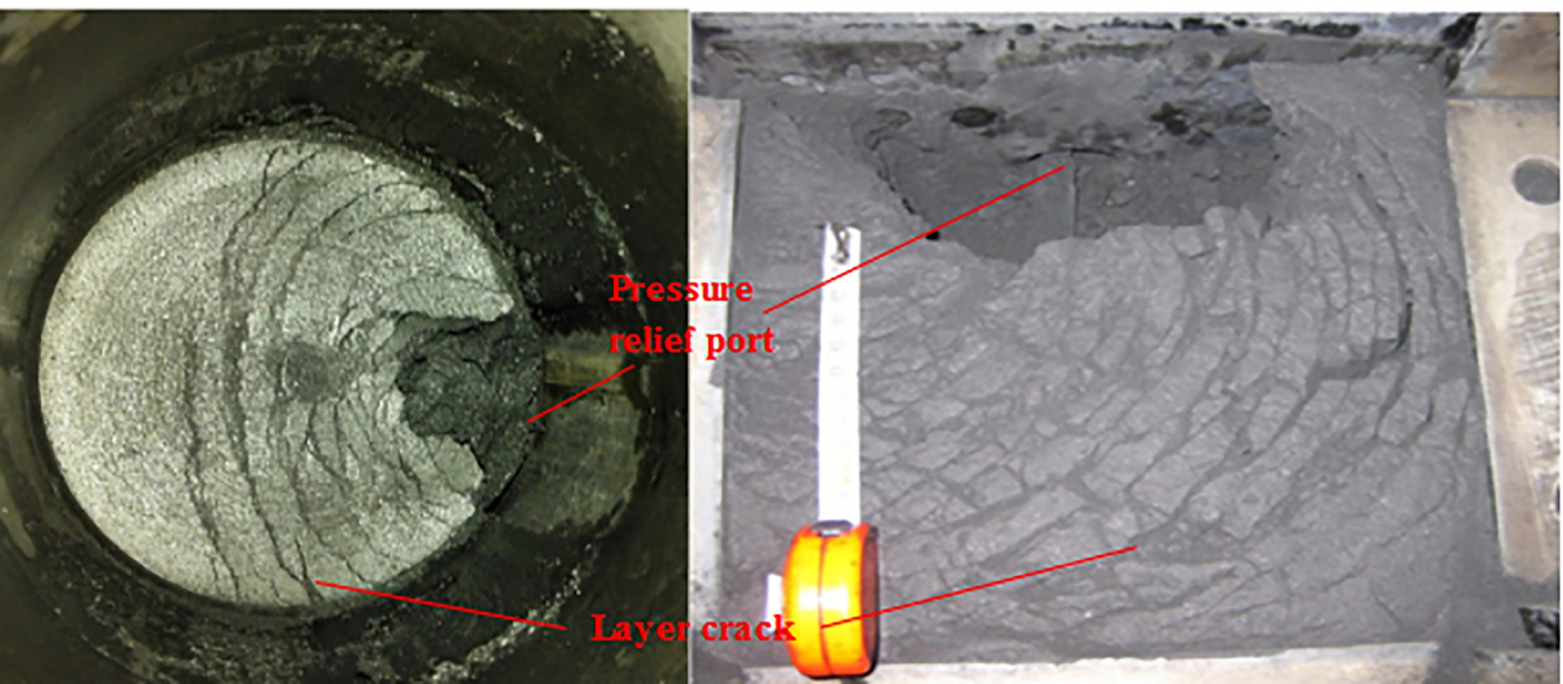
Spallation damage of gas-bearing coal after unloading pressure.

Coal and gas outburst is a common coal-rock dynamic disaster. When it occurs, a large amount of gas, accompanied by broken coal, suddenly pours into the working faces of the mines. The rapidly released energy can cause serious damage to both workers and production equipment in the mining space [1]. The complexity and nonconsensus of its occurrence mechanism is the fundamental reason why coal and gas outbursts are frequent and difficult to effectively control. Many studies have been conducted by scholars to explore the mechanism of coal and gas outburst, and many valuable research results were obtained. Most studies believe that although coal and gas outbursts are complicated, the gas pressure, ground stress, and comprehensive effect are dominant, which constitute the three main types of coal and gas outbursts. Meanwhile, the "spallation phenomenon" was discovered as a typical form of the coal and gas outburst dominated by the gas pressure, as shown in Fig 1[2, 3]. Therefore, taking the internal mechanism of spallation damage as a breakthrough point, to quantify the mechanical conditions of spallation and spallation propulsion, the spallation phenomenon can be evaluated in an attempt to further reveal the mechanism of coal and gas outbursts.

The phenomenon of spallation in gas-bearing coal after gas pressure relief was observed in the course of experiments conducted by scholars as early as the 1950s. Since then, the nature of spallation has been continuously studied by scholars. According to experimental phenomena, Khristianovich S A et al. [4] believe that the destruction of coal after the pressure relief is deeply transmitted in the form of waves into the coal; then, the coal is stripped layer by layer. Based on this study, the theory of crush wave was proposed by Khristianovich S A. Then, the Phase Transition rarefaction wave theory was proposed using Litwiniszyn J [5] method based on visual gas and coal as a miscible medium. The elastic energy release pulse was experimentally monitored by Hargraves A J et al. [6]. This type of pulse basically corresponds to the unloading waves generated by various spalls in the protrusion process. Chinese scholars Yu Shanbing et al. [7–9] also believe that the occurrence of spallation is caused by a form of wave (pulse). A simplified one-dimensional model of spallation and advancement was built on the basis of experimental theoretical research by Yu. Then, a three-dimensional model of spallation was built on the basis of a one-dimensional model by Chen Li [10]. Based on those facts, Jin et al. [11–13] established that the spallation damage is the main form of damage to the coal mass after pressure relief. Moreover, they recognized that the stress wave effect is the main cause of the spallation phenomenon caused by both rock burst and sudden pressure relief. These studies support that the pressure relief wave is the cause of the spallation failure.

However, some scholars believe that the occurrence of spallation damage is caused by the change in support stress after the pressure relief. The loading state of coal samples changes from three axes to a single axis after the pressure relief, and the stress concentrates in the vertical direction, which causes higher tensile stress in the horizontal direction and leads to the subsequent expansion and penetration of the internal fissure and spallation failure. This theory is widely recognized in the field of rockburst [14]. Spallation instability models are built on this theory [15–16]. In the field of coal and gas outbursts, the spherical shell instability theory of Jiang et al. [17] is the representative result of using support stress to explain the occurrence of spallation.

The supporting stress and effect of stress wave were regarded as the main reason for the occurrence of spallation. However, less consideration was given to the effect of the presence and flow of gas in coal on spallation. However, tensile stress will appear with the appearance of the flow field, and the tensile stress participates in the destruction and handling of the coal. The mechanical effect of the flow on porous media is well studied in the field of water inrush. The failure characteristics of coal and rock mass based on seepage instability theory was systematically studied by D. Ma et al. using experimental and numerical simulation methods [18–21]. From the perspective of seepage, the contribution of the tensile stress in the flow field to the cracking of the coal wall was studied by Paterson L [22]. In view of the damage effect of gas on the coal body during the infiltration process, Fedorchenko I A et al. performed a further study. The research findings show that free gas can destroy the coal after rapid pressure relief. The desorption of gas only plays the role of carrying the coal in the later period [23, 24]. Based on the consideration of coal adsorption characteristics, the mechanism of adsorption-desorption behavior on the evolution of flow field is deeply analyzed by Wang J G et al [25–27]. To further explore the role of gas flow and its existence in the process of spallation damage and propulsion, the effect of the flow field on the change in stress field and the contribution to the spallation after pressure unloading were studied by means of numerical simulation. The mechanical conditions for the initial damage and propulsion of the spallation are obtained based on the simplified model in this paper.

## Simulation experiment of spallation

### Brief introduction of the experimental equipment

**Fig 2:**
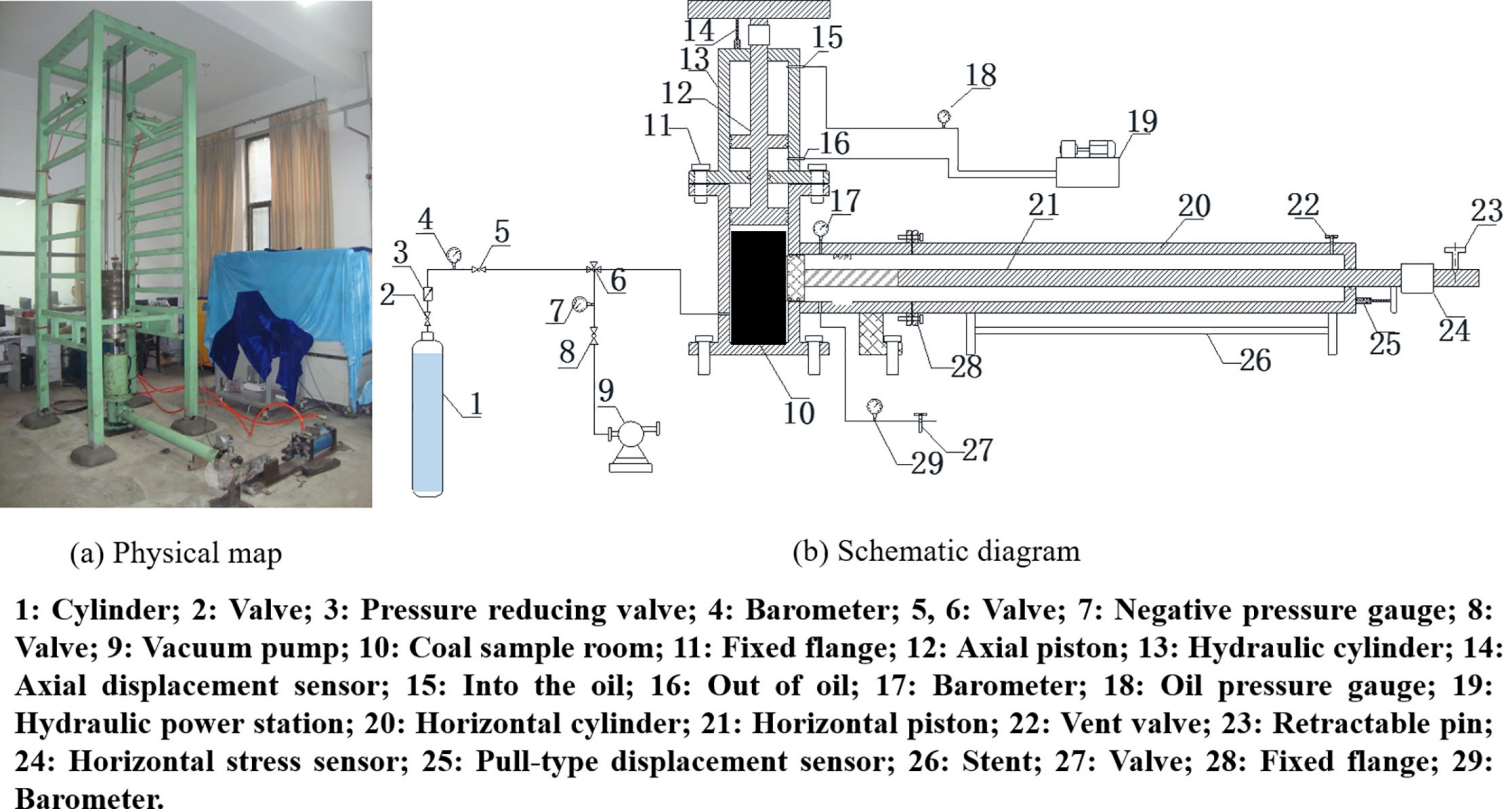
Spallation damage and outburst simulation experiment device.

A homemade experimental device, "Crack Damage and Outburst Simulation of Gas Bearing Coal Body", was used to explore the response characteristics of spallation to the experimental variables, such as loading—pressure relief, and gas pressure. The device is mainly composed of a coal sample chamber (*Φ*100 mm×L200 mm), a stress loading system, a gas supply device, a pressure unloading device and a data acquisition system; the physical drawings and schematics are shown in Fig 2.

A hydraulic cylinder with a maximum load of 150 MPa is installed on the cylindrical coal sample room (10) to load pulverized coal and apply an axial load. The coal sample room has a horizontal opening connected to a piston cylinder (20) and piston rod (21) via a flange device. The rapid pressure relief of coal samples in the horizontal direction by the rapid retraction of the fixing pin (23) is controlled by the cylinder. The high-pressure gas cylinder (1) is used to provide high-pressure gas, which will be adsorbed in the coal samples.

### Experimental scheme and simplified experimental procedure

#### Simplified experimental procedure

**Fig 3:**
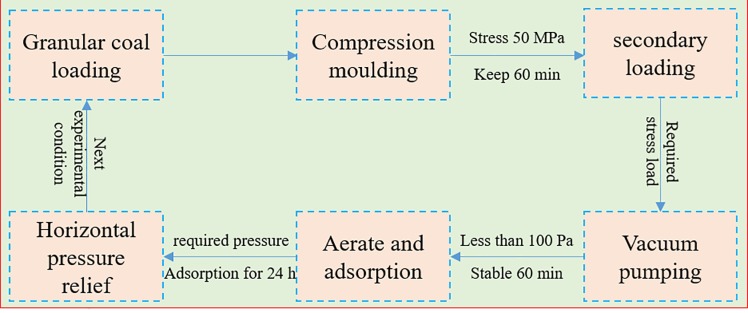
Experimental flow chart.

The whole experimental process is shown in Fig 3.

#### Experimental scheme

To explore the control effect of the stress state and flow field in the spallation failure process, simulating experiments of spallation failure under different adsorption pressures and stress conditions were performed. The specific experimental scheme is shown in Table 1.

**Table 1:** Experimental scheme.

Axial Stress (MPa)	Adsorption Pressure (MPa)
0	0, 0.6, 0.65, 0.70, 0.73, 0.78, 0.9
5	
10	

### Experimental results

Table 2 shows the experimental results of whether spallation occurred in the coal body after the pressure relief under various experimental conditions. “Yes” indicates that a spallation failure occurred after a pressure relief.

**Table 2:** Experimental results of the spallation.

		Adsorption equilibrium pressure(MPa)						
		0	0.60	0.65	0.70	0.73	0.78	0.90
Axial stress (MPa)	0	No destruction	YES	YES	YES	YES	YES	YES
	5	No development(rib spalling)	YES	YES	YES	YES	YES	YES
	10	No development(rib spalling)	YES	YES	YES	YES	YES	YES

**Fig 4:**
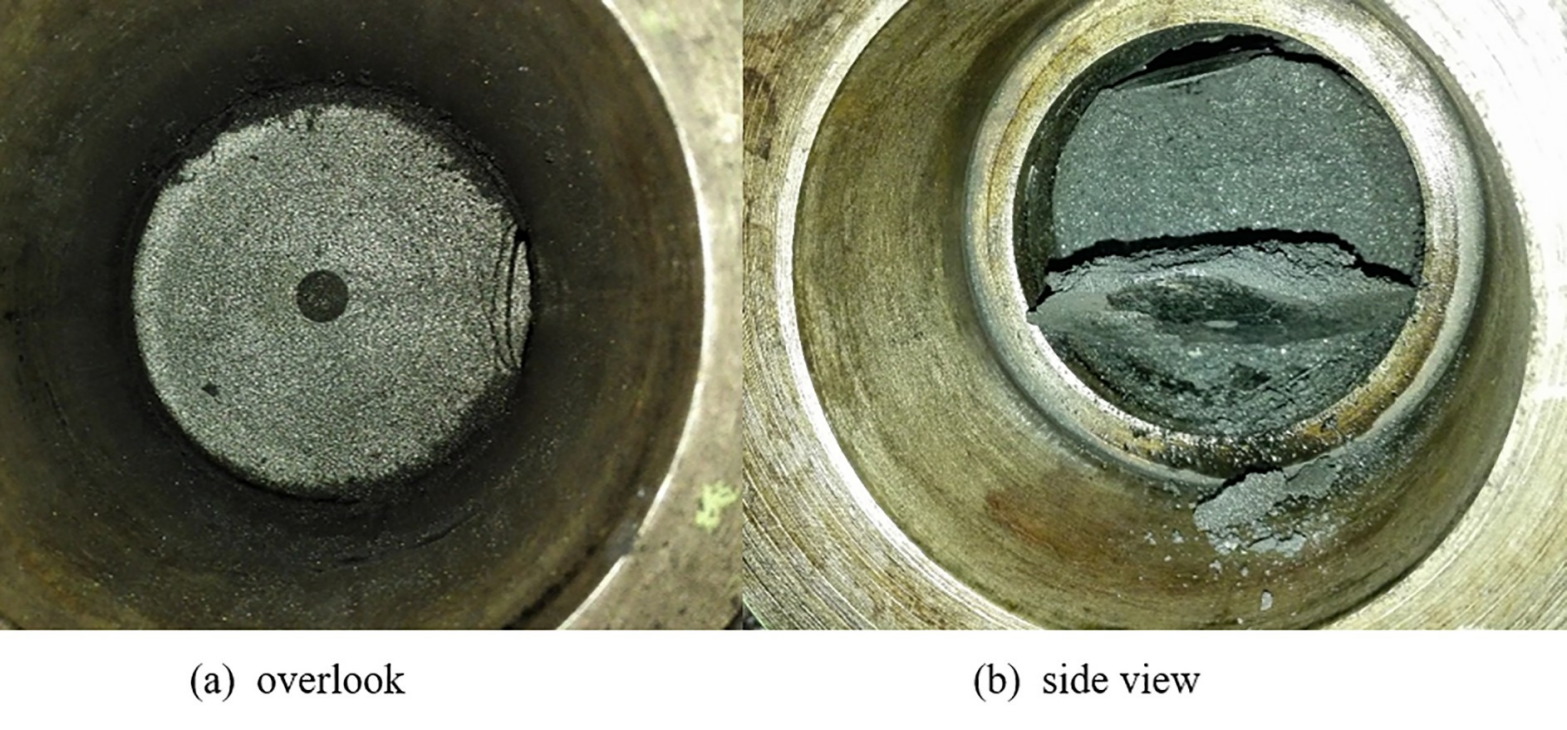
Damage phenomenon after pressure relief in the case of non-adsorbed gas.

**Fig 5:**
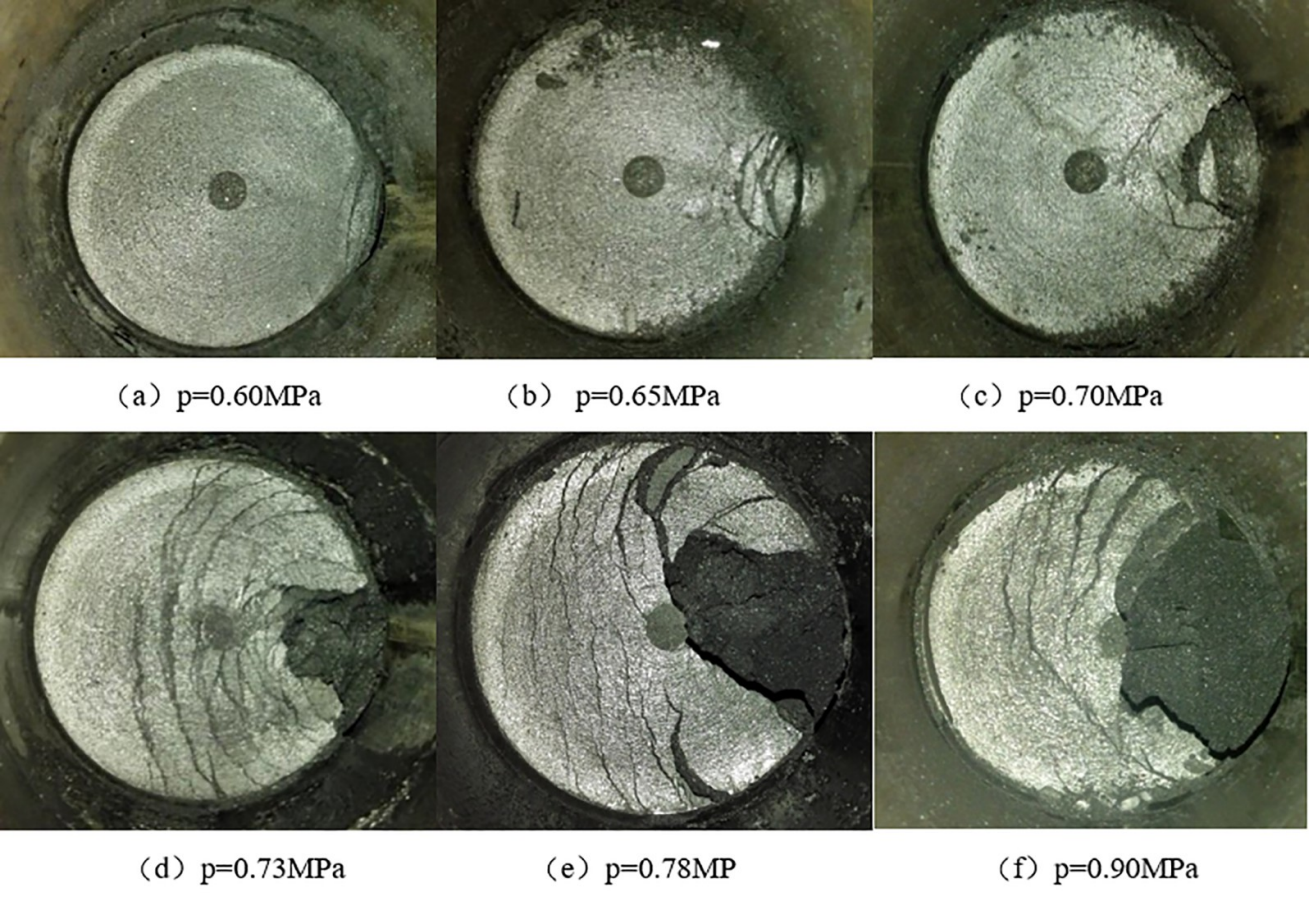
Damage degree of the coal samples under different adsorption pressures after rapid pressure unloading.

The experiment results show the following:

(1) The damage occurred only near the pressure relief port and did not advance into the deep coal body under the condition in which the stress load is applied but no gas is adsorbed. The damage is shown in Fig 4.

(2) When the size of the external load is fixed, the degree of damage of the gas-containing coal bodies after pressure relief is positively correlated with the initial adsorption pressure. The damage conditions are shown in Fig 5.

The experimental results show that spallation failure advances deep into the coal body can occur only if there is gas. The gas adsorption equilibrium pressure is the main controlling factor that can cause the spallation failure and determine the depth of spallation and destruction (degree of destruction). Therefore, exploring the damaging effect of the gas flow on coal is significant and necessary to study the characteristics of spalling failure after the gas pressure relief in gas-bearing coal bodies and to reveal the inherent spalling-failure mechanism.

## Mathematical model

**Fig 6:**
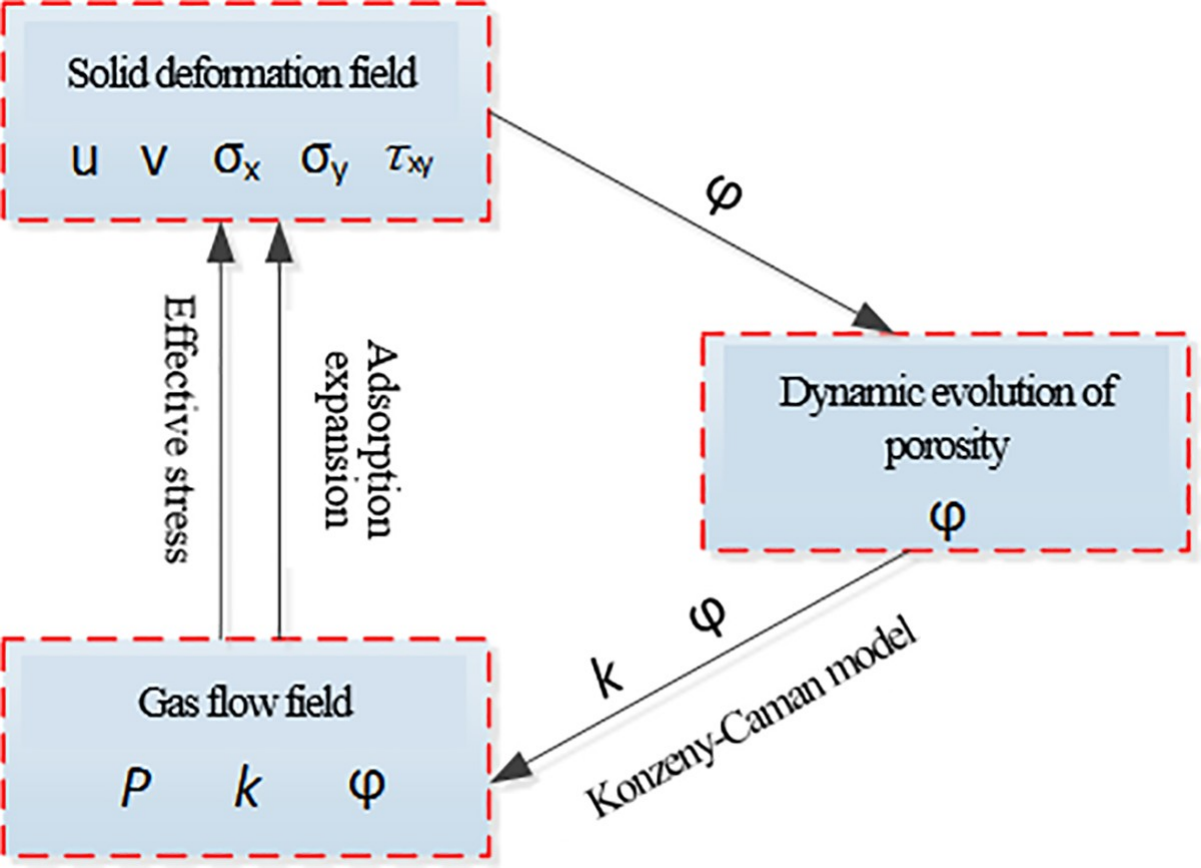
Fluid solid coupling relationship in gas-containing coal.

When the pressure is suddenly relieved in a certain direction, the original equilibrium state of the gas-containing coal is broken: ① The original stress state of the coal body will change; and ② The gas seepage field will provide a drag force to the coal body in the flow direction (tensile stress in the flow field). Therefore, the existence of the flow field will change the stress-strain state of the coal-rock medium. In contrast, the change of the strain state of the coal medium affects the seepage state of the gas. The coupling relationship between the stress-strain state and the seepage state of the gas is shown in Fig 6. In this paper, a one-way coupled mathematical model is constructed to numerically analyze the mechanical response of the coal body during gas flow while neglecting the effect of the coal deformation on the flow field.

### Basic hypothesis

The gas storage in the coal body is mainly in two states: adsorption state and free state. These states comply with the Langmuir adsorption model and ideal gas state equation, respectively:

$$C=\frac{abcpp_{n}M}{(1+bp)RT}$$ (1)

$$\rho =\frac{pM}{RT}$$ (2)

where *C* is the mass of gas in the adsorption status in a unit volume of coal, kg/m^3^; *a* and *b* are the adsorption constants, m^3^/kg and Pa^-1^; *c* is the mass of combustibles per unit volume of coal, kg/m^3^; *R* is the universal gas constant, 8.3145 J/ (kg·K); *T* is the temperature of the coal body, K; *ρ* is the density of free gas, kg/m^3^; P_n_ is the atmospheric pressure under standard conditions, Pa; *p* is the free gas pressure, Pa; *M* is the molar mass of gas, kg/mol.

The adsorbed gas does not flow and starts to desorb when reaching the critical pressure. The flow of free gas in pores follows Darcy's law as follows:

$$v_{i}=-\frac{k_{i}}{\mu }\nabla p_{i}$$ (3)

where *v*_*i*_ is the seepage vector of free gas; *k*_i_ is the permeability, m^2^; *μ* is dynamic viscosity, Pa·s.

The coal-rock medium in this study is assumed to be homogeneous and isotropic. Meanwhile, the deformation is linearly elastic before the damage occurs.

### Flow equation

According to the law of conservation of mass, the change in gas quality in the microelement is:

$$\frac{\partial m}{\partial t}=-\nabla \left(\rho \cdot v\right)$$ (4)

By substituting Eq (2) into Eq (4) and collating, the following is obtained:

$$\frac{\partial m}{\partial t}+\frac{M}{RT}\nabla \left(P\cdot v\right)=0$$ (5)

The mass of gas contained in a unit volume of coal when the pore pressure is P can be obtained according to Eq (1) and Eq (2),

$$m=\frac{abcPP_{n}M}{\left(1+bP\right)RT}+\varphi \frac{PM}{RT}$$ (6)

By combining (5) and (6), we obtain:

$$\frac{\partial }{\partial t}\left(\frac{abcPP_{n}}{\left(1+bP\right)}+\varphi P\right)+\nabla \left(P\cdot v\right)=0$$ (7)

According to the basic assumption, the flow of gas in the coal follows Darcy's law. By substituting Eq (3) into Eq (7), we obtain:

$$\frac{\partial P}{\partial t}\left[\frac{abcP_{n}}{{\left(1+bP\right)}^{2}}+\varphi \right]+P\frac{\partial \varphi }{\partial t}-\frac{1}{\mu }\nabla \left(k\cdot P\cdot \nabla P\right)=0$$ (8)

To simplify the calculation on the premise that the problem can be explained, we assume that the coal porosity and permeability are constant before the coal is damaged. The above formula can be simplified as,

$$\frac{\partial P}{\partial t}\left[\frac{abcP_{n}}{{\left(1+bP\right)}^{2}}+\varphi \right]-\frac{k}{\mu }\nabla \left(P\cdot \nabla P\right)=0$$ (9)

### Deformation equation

Based on the above assumptions and the theory of elastic mechanics deformation, the deformation equation and equilibrium equation of coal are as follows,

$$\varepsilon_{ij}=\frac{1}{2}(\mu_{i,j}+\mu_{j,i}),(i,j=1,2,3)$$ (10)

$$\sigma_{ij,j}+f_{i}=0$$ (11)

where *ε*_*ij*_ is the component of the strain tensor, *u*_*i*,*j*_ is the displacement component, *σ*_*ij*,*j*_ is the component of the stress tensor, *f*_*i*_ is the component of body force, and *i*,*j* represent the space coordinates.

According to the theory of elasticity of porous media, the constitutive equation of the coal considering the adsorption is [28],

$$\varepsilon_{ij}=\frac{\alpha }{3K}p\delta_{ij}+\frac{\varepsilon_{s}}{3}\delta_{ij}+\frac{1}{2G}\sigma_{ij}-\left(\frac{1}{6G}-\frac{1}{9K}\right)\sigma_{kk}\delta_{ij}$$ (12)

where the expansion deformation of coal due to adsorbed gas is [29],

$$\varepsilon_{s}=\frac{4ac\rho_{v}RT\ln\left(1+bp\right)}{9V_{m}K_{s}}$$ (13)

Combining Eqs (10), (11), (12) and, (13), we obtain a Navier-type deformation control equation of coal seam

$$Gu_{i,kk}+\frac{G}{1-2v}u_{i,ki}-\alpha p_{i}-K\frac{4ac\rho_{v}RT\ln\left(1+bp_{i}\right)}{9V_{m}K_{s}}+f_{i}=0$$ (14)

where *E* is the elastic modulus; *G* is the shear modulus; *σ*_*kk*_ is the normal stress component; *δ*_*ij*_ is Kronecker symbol; *α* is Biot coefficient of the pore matrix, *α* = 1−*K*/*K*_*s*_, *K* is the bulk modulus of coal, and *K*_*s*_ is the bulk modulus of the coal matrix; *ρ*_*v*_ is the apparent density of coal; and *V*_*m*_ is the molar volume of gas.

## A base model

**Fig 7:**
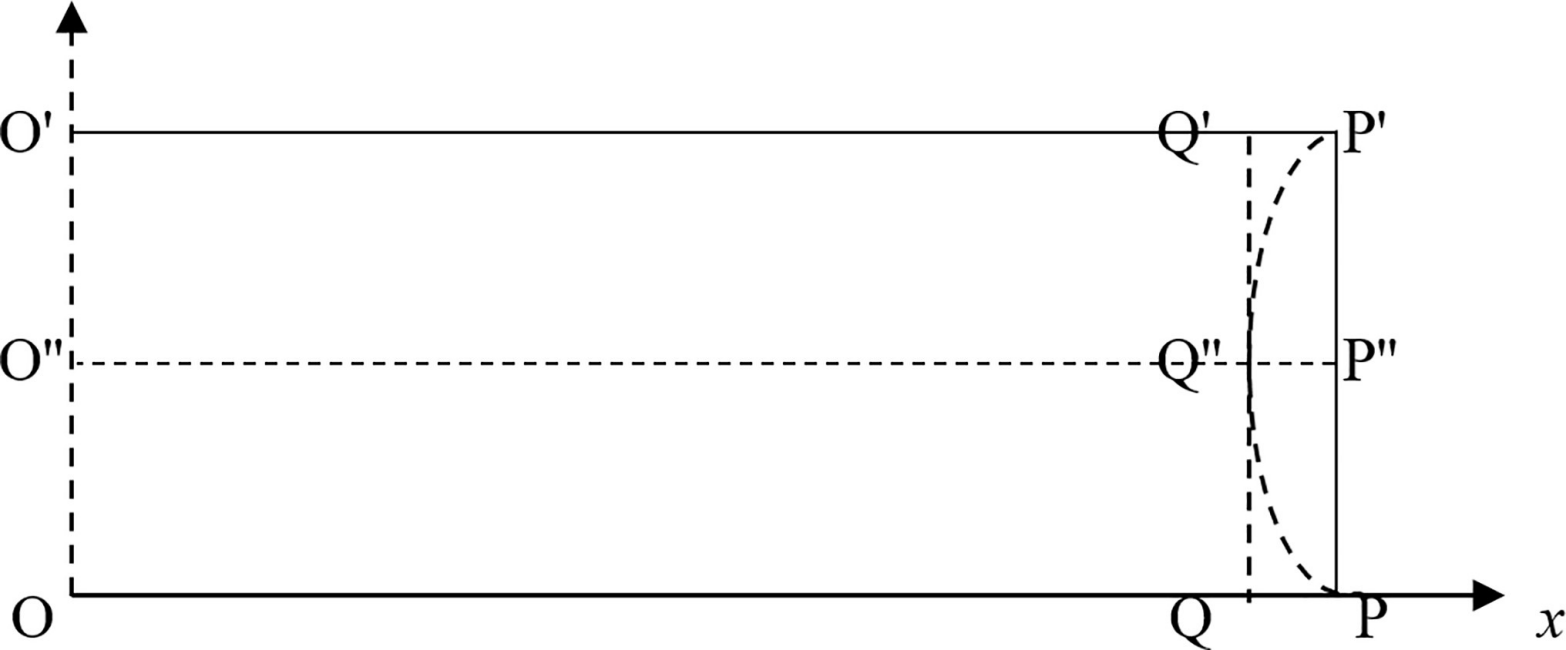
Schematic diagram of the geometric model.

A simplified two-dimensional plane model was constructed by using COMSOL Multiphysics in this study, as shown in Fig 7. O′P′ and OP are the roof and floor of the coal seam, respectively. Since this study focuses on the gas pressure distribution and its effect on coal in the process of gas seepage, the effect of the ground stress on the stress and strain of coal is ignored in this model. PP′ is the pressure relief surface exposed after mining; OO′ is the symmetrical boundary of deep coal seam. OP is the zero-flux and fixed boundary; QQ′ is a virtual cutting plane inside the coal seam. O′P′ is zero-flux, and its X-direction displacement is limited. When *t* = 0, the equilibrium adsorption pressures in the solution domain are: 0.8 MPa, 1.2 MPa, 1.5 MPa; when t>0, *PP*′ is the free-pressure-relief boundary.

By being able to explain the failure mechanism of spallation, the relevant parameters in the fluid-solid coupling model of the gas-bearing coal-bearing rock are selected, as shown in Table 3.

**Table 3:** Simplified model calculation parameters.

Name	Bulk moduli	Matrix moduli	density of coal	density of gas	dynamic viscosity	Adsorption Volume Constant	Adsorption pressure constant	Poisson ratio
Numerical value	1.5E+9	5.76E+10	1450	0.714	1.08E-06	0.036656	1.12E-06	0.34
Dimension	Pa	Pa	kg/ m^3^	kg/ m^3^	Pa·s	m^3^/ kg	Pa^-1^	1

## Numerical solution results and analysis

**Fig 8:**
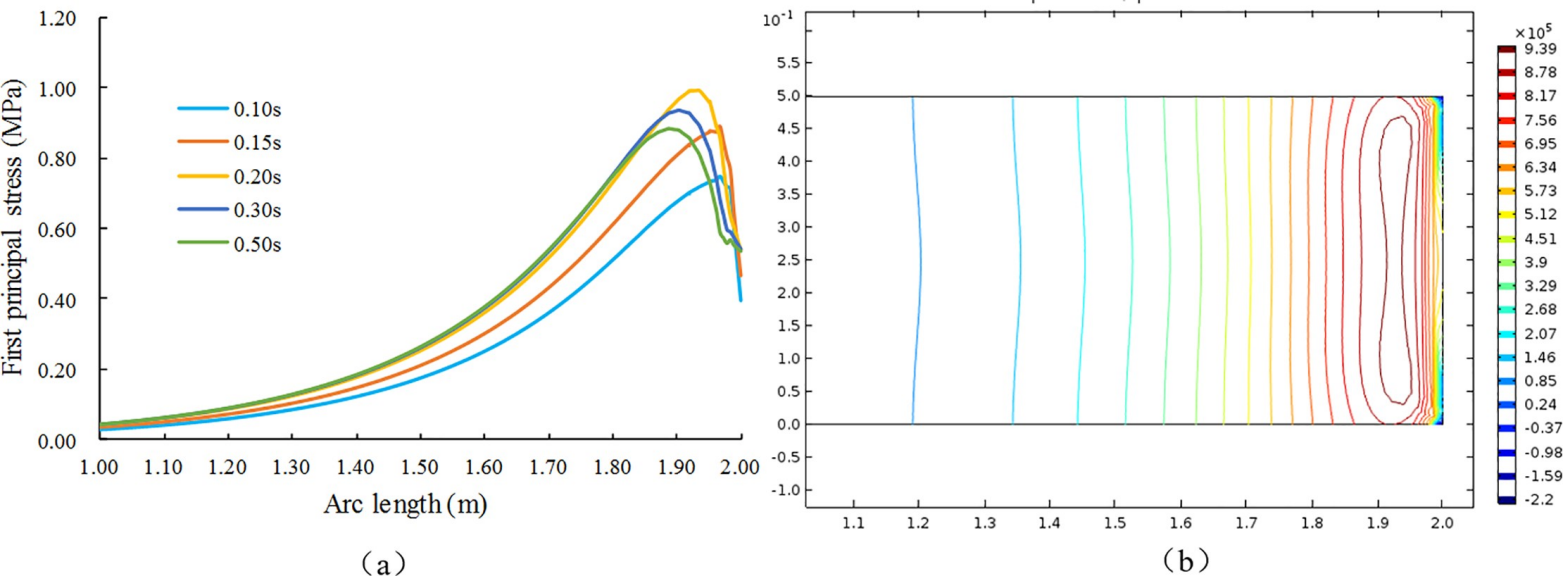
Distribution of the first-principal stress in the X direction and distribution of regional contours at t = 0.1 s.

When the adsorption equilibrium pressure is 1.2 MPa, the tensile stress curve over time and the contour distribution in the solution domain at 0.1 s on the model line O″P″ is shown in Fig 8.

Fig 8(A) shows that the first-principal stress on the X axis is tensile stress, and its peak appears at a certain distance inside the pressure relief surface. When the tensile stress peak at a certain moment is greater than the tensile strength of the coal body, the tensile failure of coal will occur at this moment, and a layer with a certain thickness is formed. Meanwhile, Fig 8(B) shows that the tensile stress concentration appears on both sides of the pressure relief surface. When damage occurs, the destruction of the crack will propagate along the direction of the minimum principal stress (arc), which results in a macroscopic fracture, as shown in Fig (7), and the arched layer-crack structure is broken along P′Q″P′. This phenomenon is caused by the fixed boundary conditions of the roof. If the roof and floor of the coal seam are free sliding boundaries, the first damage will occur along the line QQ″ in Fig (7), and a layer-crack structure with uniform thickness will be stripped.

### First spallation process

#### Analysis of the evolution process

**Fig 9:**
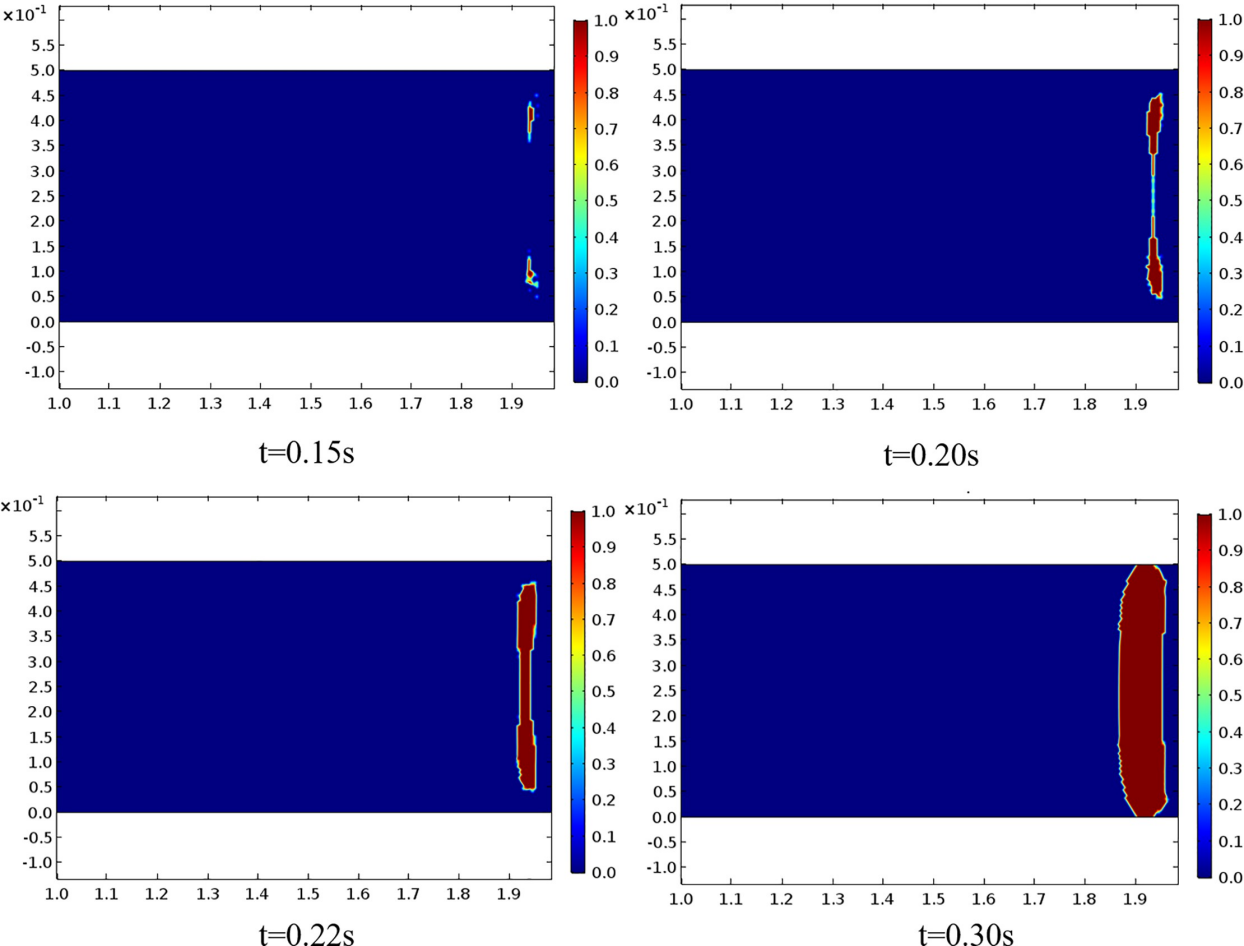
Regional evolution of the maximum first-principal stress above the tensile strength in the solution domain.

If we assume that the tensile strength of coal body is 0.8 MPa, the coal may be damaged in the area where the first-principal stress is greater than 0.8 MPa. The evolution process of the area where damage may occur is shown in Fig 9.

Fig 9 shows that the area in which the first-principal stress is greater than the tensile strength appears at 0.15 s and expands from the roof and floor to the middle part over time; at t = 0.20 s, the failure area was basically penetrated from the upper and lower ends to the middle. The thickness of the layer-crack structure was determined as it was stripped. The initial destruction was basically completed at this time. Because the solution is based on linear elastic media, the entire process consists only of stress, and no damage can occur.

#### Determination of quantitative conditions

**Fig 10:**
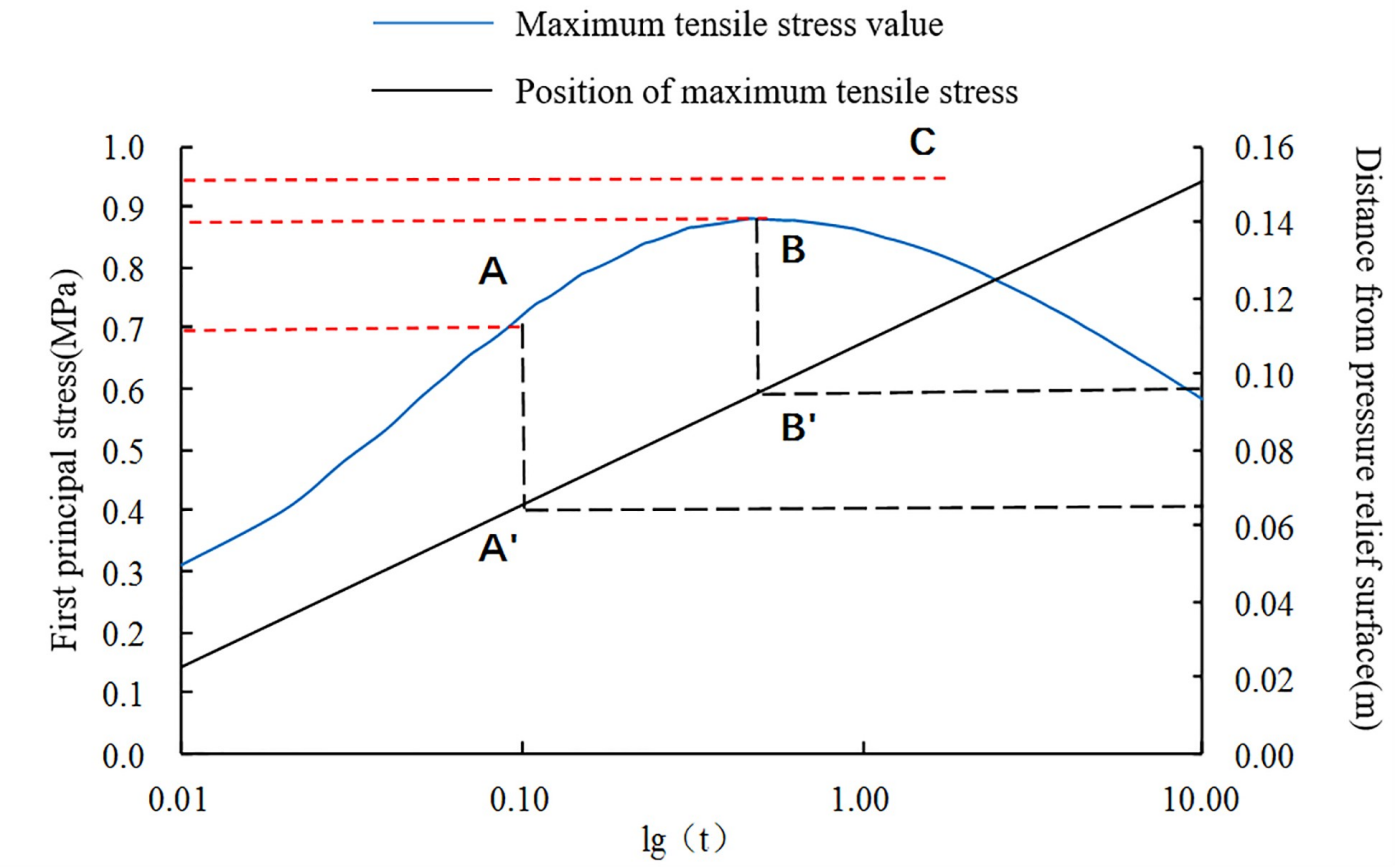
Simulation results of the maximum tensile stress and its position at different times after unloading.

Each parameter of the flow field is a continuous function of time in the entire solution time domain. Therefore, the tensile stress caused by the flow field is also a continuous function that changes with time, as shown in Fig 8(A). Furthermore, the peak of the tensile stress and its location are continuous functions with respect to time (*t*). The calculation data of the tensile stress peaks and peak positions are extracted and organized, and the time data are processed using logarithmic coordinates. The relationship between the two and time is shown in Fig 10.

Whether spallation can occur, as well as the location of the initial spallation, can be obtained from Fig 10. The calculation shows that the peak of the tensile stress in the direction of flow after pressure relief first increases and then decreases in the time domain. The position of the peak appears to gradually extend deeper into the coal body over time, and it has a linear relationship with time. Whether spallation can occur depends on the tensile strength Rm of the coal body itself.

1) Rm = C: The coal will not be damaged because the peak of the tensile stress is less than the coal tensile strength for the entire time domain. The energy storage inside the coal is slowly released by the seepage and does not participate in the destruction and throwing of coal;

2) Rm = B: The tensile strength is exactly equal to the maximum tensile stress peak in the time domain, so the coal medium may be destroyed at B'. The projection value (0.095 m) of B' on the secondary coordinate is the position where the splitting damage may occur. However, the spalling will not advance into the depth of the coal body;

3) Rm = A: The time when the primary spallation failure occurs is advanced, and the thickness of the layer-crack structure is reduced. In this case, spalling may continue to advance.

Therefore, the results demonstrating whether spallation can occur, as well as the thickness of layer-crack structure, are controlled by the tensile strength of the coal when the adsorption equilibrium pressure is constant.

**Fig 11:**
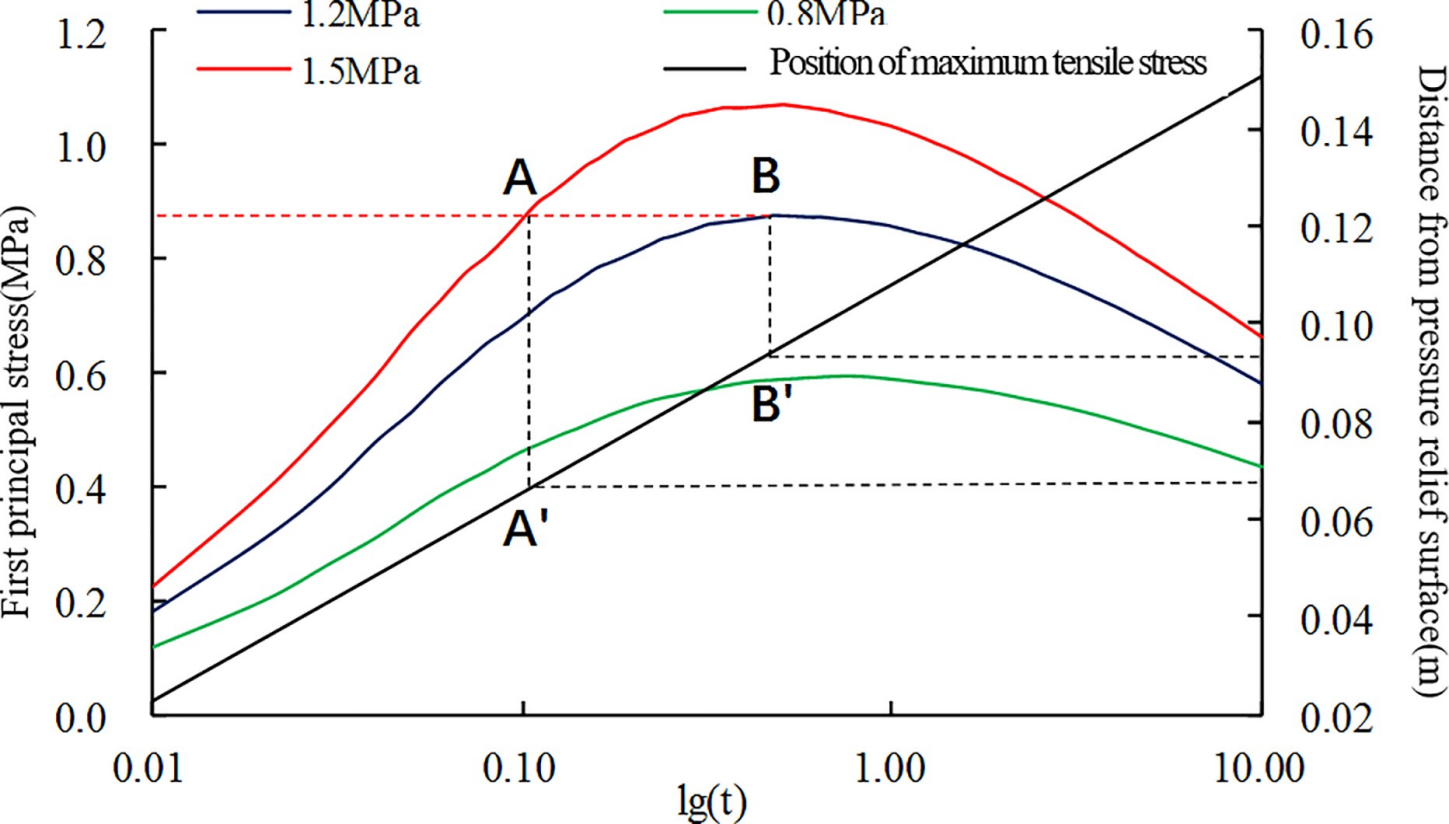
Maximum tensile stress and its location with time under different adsorption pressures.

Similarly, the numerical calculation data of three groups of adsorption equilibrium pressure conditions are extracted. We obtain their quantitative relationship, as shown in Fig 11.

The calculation results show that the locations of the maximum tensile stress of the three groups of data are basically identical, but the peak tensile stress values in the coal have obvious differences. It is assumed that the tensile strength of the coal body is R_m_ = AB:

1) *P* = 0.8 MPa: No spalling occurs because the maximum tensile stress generated in the entire time domain is less than the tensile strength of the coal;

2) *P* = 1.2 MPa: The peak value of the maximum tensile stress in the entire time domain is equal to the tensile strength of coal. Coal may be destroyed at B', and the projection coordinates of point B' on the secondary coordinates (0.095) provide the thickness of the layer-crack structure. However, the spalling will not advance into the deep coal body;

3) *P* = 1.5 MPa: The tensile stress generated inside the coal after the pressure relief is equal to the tensile strength of the coal at point A´. A shorter time is required for the initial damage, and the layer-crack structure is thinner. In this case, spalling may continue to advance.

Thus, a higher adsorption equilibrium pressure corresponds to a smaller thickness of the layer-crack structure, which is more conducive to the promotion of spallation failure.

### Continuous promotion mechanism and termination conditions of spallation

**Fig 12:**
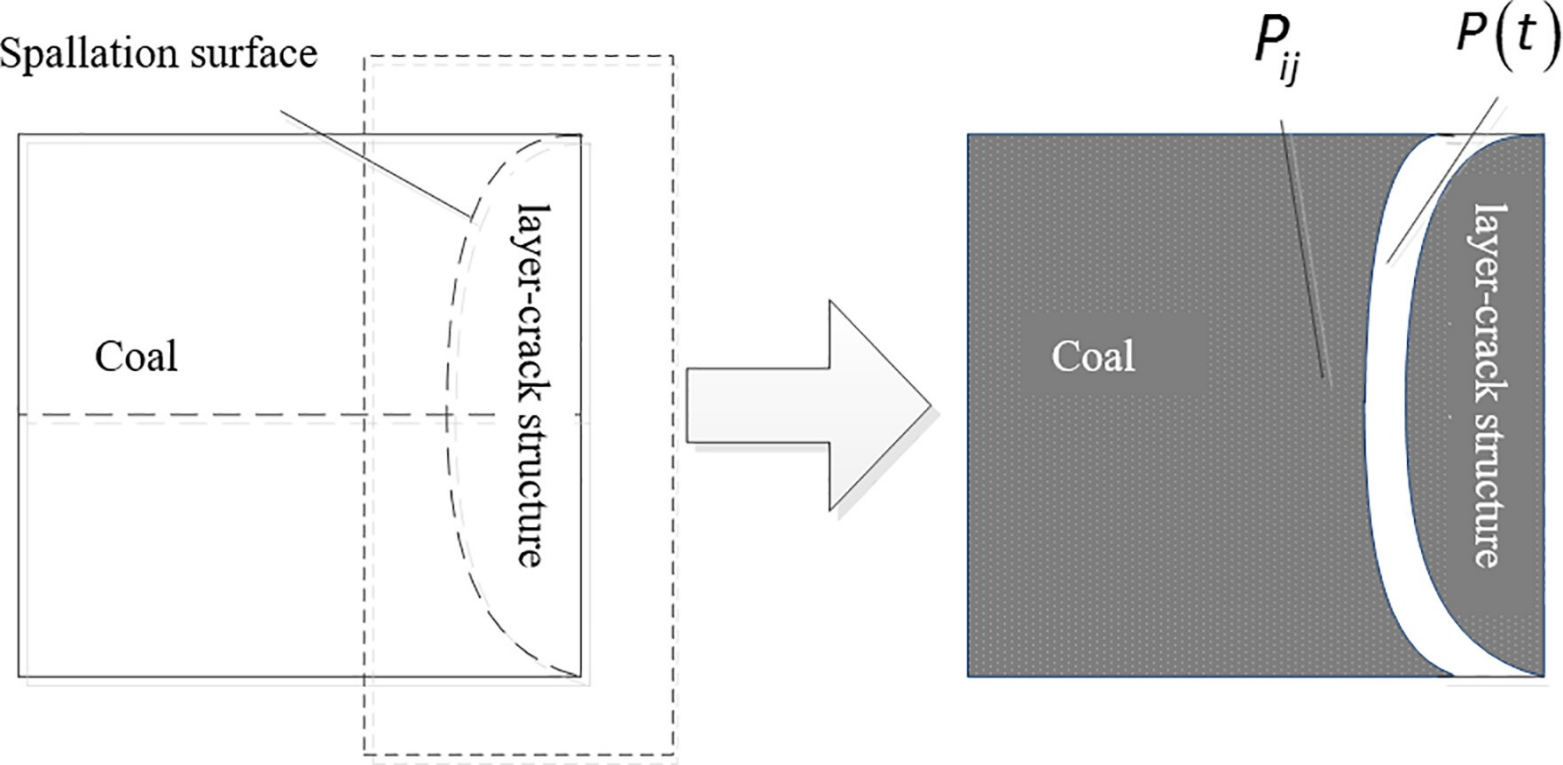
Schematic diagram of the initial spallation of coal.

After the initial destruction has occurred, the internal stress of the coal body is redistributed due to the appearance of a new pressure relief surface. The new stress peaks and their locations are also functions of time. The newly generated tensile stress peak is larger than the tensile strength of the coal body and controls whether the spallation damage can continue to occur. Then, the newly generated tensile stress peak and its position are mainly controlled by two factors: *P*_*ij*_ (gas pressure distribution in the solution domain (coal) after the initial destruction) and *P*(*t*) (pressure drop of boundary conditions), as shown in Fig 12.

#### Distribution of gas pressure in coal after the first destruction

**Fig 13:**
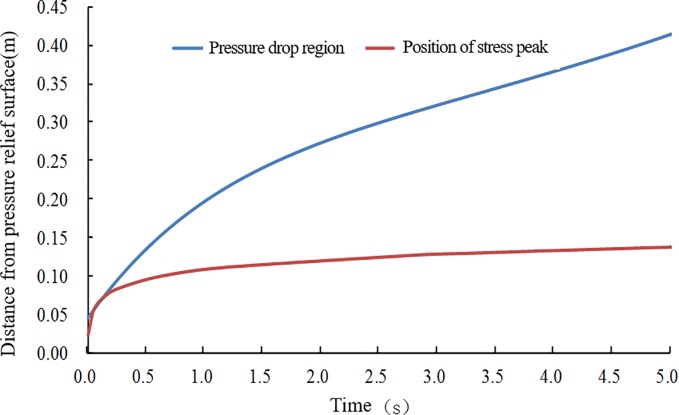
Calculation results of the peak tensile stress and pressure relief range at different times.

Numerical calculation data are extracted and organized. We obtain the numerical results of the seepage pressure drop range and tensile stress peak position, as shown in Fig 13.

In Fig 13, the tensile stress peaks appear in the pressure relief range at any time step throughout the time domain. The propelling speed of the failure surface to deep coal is smaller than the expansion speed of the gas seepage area. Therefore, compared with the initial destruction process, the maximum pressure gradient and effective stress peak that appear in coal will be reduced, even if the pressure relief rate of the pressure relief boundary is consistent with the initial pressure relief, which is determined by the initial gas pressure in the coal. Therefore, the failure ability of flowing gas to coal is constantly decaying in the entire process. In other words, the failure level of spallation is controlled by the initial adsorption pressure, mechanical properties of coal, stress state and boundary pressure relief rate. However, spallation will eventually terminate and will not continue to push forward indefinitely.

#### Discussion on the influence factors of the pressure relief rate of the lamination space boundary

**Fig 14:**
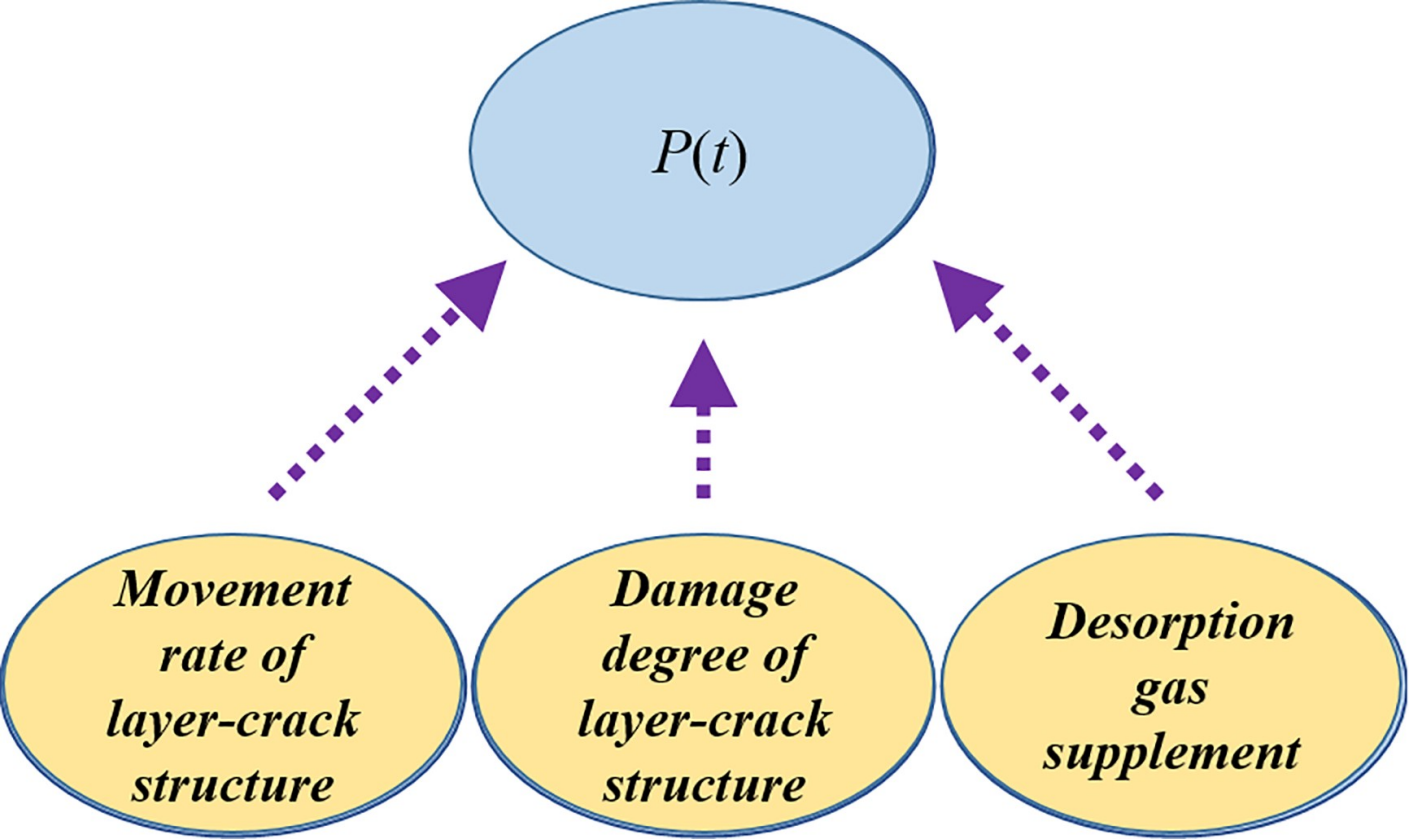
Factors that affect the variation of the gas pressure in the spallation space.

As the boundary condition of the evolution of the internal stress, the pressure change *P*(*t*) in the spallation space determines the size of the tensile stress in coal and whether the damage can continue to advance. The change in gas pressure inside the spallation space is a very complicated process because the spallation space is an open-source space. The layer-crack structure quickly moves after being stripped, and this movement causes a rapid decrease in gas pressure in the spallation space. The decrease in gas pressure promotes the desorption of adsorbed gas and reduces the pressure drop rate. Therefore, there is a coupling relationship between them. In addition, there is damage or even smashing during the movement of the simultaneous layer cracks, which causes greater uncertainty in the boundary pressure changes. In short, *P*(*t*) is mainly controlled by factors such as the movement of the stratified layer, fragmentation, and adsorption gas desorption, as shown in Fig 14.

**Fig 15:**
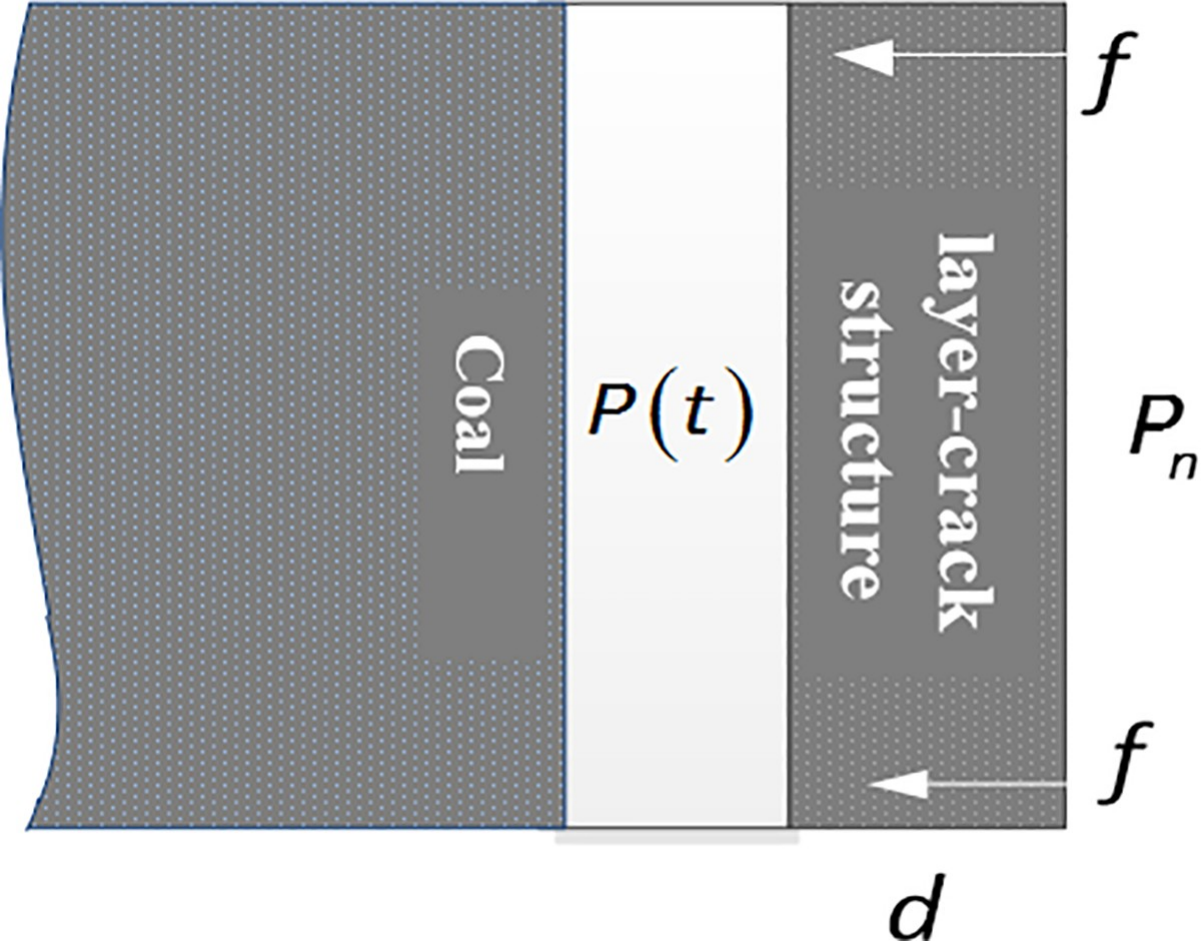
Schematic diagram of the simplified model of spallation motion.

After the spalling failure surface appears, the spalling body is in an independent state. Whether the spallation body can move and the moving speed depend on the gas pressure on both sides of the layer-crack structure and movement resistance. A simplified layer-crack structure motion model is constructed as shown in Fig 15. The front and back pressures of the layer splits are *P*(t) and *P*_n_, respectively, the thickness of the layer splits is *d*, and the movement resistance per unit length is *f*.

Fig 15 shows the conditions of the spallation movement,

$$P\left(t\right)-P_{n}\geq d•f$$ (15)

The cementation strength of coal and the cementation strength between the coal seam and the roof and floor determine the failure location and failure mode of the spallation body. It is generally believed that in the outburst-threat coal seam, the coal body has a smaller cementation strength than the coal body and the roof and floor. Therefore, the premise for the movement of the layer-crack structure after being stripped is to shear failure within the coal body and overcome the internal cohesion and friction force of the coal body. The analysis shows that the layer-crack structure forms as a result of the tensile stress failure, and the layer-crack structure moves as a result of the shearing failure inside the coal body. Therefore, in terms of failure forms, spallation failure can be promoted as a process of alternating shear stress failure. Moreover, the increase in thickness *d* of the layer-crack structure hinders its shear failure and movement. Therefore, a thinner theoretical upper layer is more conducive to the continuous advancement of spallation and destruction.

It is difficult to make a conclusion in the respect that the degree of fragmentation of the layer-crack structure affects the variation of gas pressure in the spallation space. On one hand, the breakage of the layer-crack structure aggravates the massive desorption of the adsorbed gas in the interior of the layer, which has a supplementary effect on the gas in the spallation space, which decreases the pressure drop rate in the space and hinders further expansion of the spallation. On the other hand, the breakage of the layer-crack structure can provide a fast release channel for the gas in the layer space, which accelerates the pressure drop, thus promoting the continued occurrence of the spallation failure and advancement to the deep coal. Thus, further research must be conducted in the future.

## Conclusions

(1) The gas flow plays a crucial role in the occurrence of spallation and destruction of coal after pressure relief. The formation of a layer-crack structure results from the tensile stress failure, while the movement of the layer-crack structure results from the shearing failure inside the coal body. In terms of failure form, spallation failure can be promoted as a process of alternating shear stress failure.

(2) Whether spallation can occur and where it occurs are determined by the initial gas pressure, tensile strength of coal, and pressure relief rate at the boundary. The thickness of the layer-crack structure is positively correlated with the initial adsorption gas pressure and boundary pressure relief rate and is negatively correlated with the mechanical strength of coal. A thinner layer-crack structure is more conducive to the advancement of the splitting damage to the deep part of the coal.

(3) The continuous advancement of splitting damage is mainly determined by the pressure relief state of the undamaged coal and pressure variation in the spallation space. The former can be quantified by numerical calculation; the latter is related to the thickness of the layer-crack structure and its degree of fragmentation when it moves. It cannot be quantitatively described at present and requires further study.
